# “I got all sorts of solitude, but that solitude wasn't mine”: A mixed‐methods approach to understanding aloneness during becoming a mother

**DOI:** 10.1111/bjop.70019

**Published:** 2025-09-02

**Authors:** Thuy‐vy Nguyen, Delali Konu, Deborah Tetteh, Pearl Tshimbalanga, Julie Weissova, Mingyao Xiong

**Affiliations:** ^1^ Durham University Durham UK; ^2^ University of York York UK; ^3^ Leeds Beckett University Leeds UK; ^4^ University College London London UK

**Keywords:** development, early childhood, health, social, well‐being/happiness

## Abstract

This study examines the evolving experiences of “aloneness” in first‐time mothers during their transition to motherhood. While the term is often used to describe new mothers' experiences, it tends to blur distinct yet overlapping constructs such as solitude, loneliness, and social isolation. Study 1 involved qualitative interviews with 22 mothers, revealing three themes: the ambivalent companionship of a baby, the multifaceted nature of post‐motherhood aloneness, and a shift in priorities that diminished both the quantity and quality of solitude. Although mothers often spent more time physically alone, solitude free from caregiving demands became scarce, contributing to increased loneliness and isolation. Time alone with a baby was perceived variably, depending on interaction level and caregiving demands. Study 2 analysed one‐week Ecological Momentary Assessment data from 47 new mothers, tracking daily activities and emotional well‐being. Personal time (time spent for oneself) and social time were both linked to improved mood. These findings highlight the challenges of accessing restorative time when under sustained emotional and cognitive demands. Beyond early parenthood, this study extends solitude research by providing empirical evidence that subjective solitude is shaped not only by social presence or absence but also by the psychological load imposed by social demands.

## BACKGROUND

The transition into motherhood is a profound life event that extends beyond the well‐documented physical and physiological changes of childbirth. It involves a complex restructuring of lifestyle, identity, and priorities (Emmanuel & St John, 2010). Despite antenatal preparation, many first‐time mothers report feeling unprepared for the demands of motherhood, including changes in autonomy, social relationships, and access to personal time (Nelson, 2003). Central to these challenges is the experience of “aloneness,” often described as a common but poorly understood aspect of maternal transition (Levesque et al., 2020; Rogan et al., 1997). This term has been used in various ways: it might refer to time spent alone with the baby during the early postpartum period, or it might describe a more general feeling of loneliness or even social isolation due to the overwhelming responsibility of childcare (Barclay et al., 1997; Rogan et al., 1997). As such, the term “aloneness” is often conflated with three conceptually distinct phenomena: loneliness, social isolation, and solitude.

Loneliness refers to a subjective, often distressing feeling that one's social connections do not meet personal expectations for connections or support (Russell et al., 2012; Valtorta et al., 2016). In the context of perinatal loneliness, loneliness may be felt at an emotional (feeling a lack of connections), a social level (feeling a lack of community or broader social network), or even at an existential level (feeling deeply disconnected from others or from one's previous sense of self) (Naughton‐Doe et al., 2025). On the other hand, social isolation is an objective state characterized by limited access to social contacts (Russell et al., 2012; Valtorta et al., 2016). A mother may be socially isolated without feeling loneliness, or may feel profoundly lonely even though they are surrounded by other people. Both loneliness and social isolation are common during maternal transition, especially when mothers feel they are “going at it alone” without adequate support or connections, coupled with a sense of alienation from their identity before having the baby (De Goede & Greeff, 2016; Wilkins, 2006).

Meanwhile, solitude refers to either a physical state of being alone or a subjective experience of being detached from social demands (Long et al., 2003). Solitude can be experienced positively or negatively, depending on contexts (Lay et al., 2018; Pauly et al., 2018). Although both mothers and fathers report spending less time alone after having children compared to non‐parents (Lindberg, 2017) and having less time for themselves (Levesque et al., 2020), solitude itself does not directly contribute to negative experiences. In fact, time spent alone can allow for rest and restoration (Hammond, 2016; Nguyen et al., 2018; Rodriguez et al., 2020) and offer a sense of autonomy and freedom (Weinstein et al., 2023). On the other hand, a lack of needed solitude can be associated with distress (Coplan et al., 2019).

The existing literature acknowledges that new mothers spend more time at home and alone (Feldman et al., 2004), particularly after a partner returns to work. However, this time is rarely spent physically alone, as the baby is typically present. As such, the solitude captured in this research is more likely to be subjective rather than objective physical solitude (Campbell & Ross, 2021; Nguyen & Rodriguez, 2024). This raises an important question: does time alone with a baby count as solitude? Some of these moments may involve bonding and interaction, while others are dominated by caregiving demands. The distinction may lie in how mothers experience these moments, whether as a form of connection or as an extension of responsibility.

At the same time, the constant presence of the baby also explains why mothers report a lack of “me‐time” for self‐care or leisure (Levesque et al., 2020), suggesting that solitude as a chosen and restorative experience may be rare. Even when mothers are alone, such as during a baby's nap, they often remain psychologically preoccupied with caregiving demands. This paradox, where physical aloneness coexists with mental overload and emotional disconnection, highlights a need to examine aloneness not only as a challenge but as an evolving and multifaceted experience.

Recognizing these gaps, we conducted a two‐phase investigation to unpack the complexities of aloneness in motherhood. First, we undertook a qualitative study to explore mothers' lived experiences and understand how they conceptualized aloneness in their daily lives. This approach allowed us to capture the nuances of solitude and related experiences, such as loneliness and isolation, without imposing predefined frameworks. Mothers reflected on their time spent alone, both with and without their child, and on how these experiences had shifted since becoming a parent. By providing mothers the space to articulate their own perspectives, we sought to disentangle the subjective and objective dimensions of aloneness.

Building on these qualitative insights, we conducted a second, quantitative study to examine how specific types of aloneness—being completely alone, being alone with the child, and engaging in personal activities—relate to emotional and psychological well‐being. By integrating both qualitative and quantitative methods, our research offers a clearer understanding of aloneness during the maternal transition, highlighting its complexities and potential implications for maternal well‐being.

## STUDY 1

### Procedure

Study 1 included both one‐on‐one interviews and focus groups to gather a range of perspectives from participants. The inclusion of focus groups allowed us to take advantage of group dynamics, giving participants the opportunity to reflect on and expand their own experiences in response to what others shared (Brown, 2018). For example, one mother initially remained quiet but, after listening to the group discussion, opened up about not receiving support from her own mother.

### Interview questions

Interview questions were developed by the research team with feedback from researchers with expertise in the topic of solitude across two expert focus group panels. The questions went through two rounds of revisions to ensure they captured our research aims to understand mothers' solitude and explore the transition to motherhood in a broader sense. The research team also completed four training sessions with a qualitative expert to go over the finalized interview questions, best practices in conducting interviews, and how to engage in reflexivity exercises.

### Individual characteristics

Participants responded to an online survey hosted on Qualtrics (Version 2023; www.qualtrics.com), answering demographic questions about their age, gender, ethnicity, employment status, level of education, marital status, residence, and household status. Participants also completed questions about their physical health (Haerpfer et al., 2020), life satisfaction (Haerpfer et al., 2020), and socioeconomic status (Haerpfer et al., 2020). Additionally, we also asked each participant to estimate their daily number of hours spent in solitude. We provided them with a definition of solitude as time when they are “not interacting with other people, either in person, on the phone, or online”, and asked “on a typical day, approximately what proportion of your waking hours (i.e. excluding time when you are asleep) is spent in solitude?”. After that, they were asked whether they felt that the amount of time they spent alone each week was either “definitely not enough”, “somewhat less than [they] would like”, “just about right”, “somewhat more than [they] would like”, or “definitely too much”.

### Interview protocol

The interviews were semi‐structured and consisted of 10 open‐ended questions. First, we were interested in the mother's attitudes toward solitude prior to becoming a mother and after. Second, we asked questions about her perception of norms regarding solitude that are shaped by her close others and/or social circles. Third, we wanted to understand how much control she felt that she had over her solitary time, either when she needed that time or when solitude happened spontaneously. All participants were provided a definition of solitude, which is “times when [you are] not interacting with someone else in person, not talking on the phone or chatting with other people online” (Lay et al., 2018; Pauly et al., 2018), and were asked to consider their solitary experiences in relation to their transition into motherhood.

Interviews were conducted in English; online individual interviews were recorded via Microsoft Teams for up to 1 hr, and in‐person focus groups were recorded using a Dictaphone for up to 1.5 hr. To ensure privacy, participants could keep their camera on or off for online interviews, and those who participated in focus groups only referred to themselves or each other using their anonymous IDs. At the end of the interview, participants were debriefed, and interviewers completed reflexivity statements to document their thoughts about the participants and the interviews.

### Transcriptions

Interviews were transcribed by the research team from scratch or by editing the Microsoft Teams interview transcript. Interviews were transcribed non‐verbatim as suggested by McLellan et al. (2003). During this screening process, two transcripts were identified as poor quality due to technological issues with the recordings, and two were identified as potentially inauthentic based on interviewers' reflexivity statements. We checked the themes that were identified in those interviews at the end of data analysis and did not find any inconsistencies compared to the other transcripts; so we included all the transcripts when reporting the results.

### Data analysis

Thematic Analysis (Braun & Clarke, 2006) was used to identify salient themes and codes related to early motherhood and solitude. Four members of the research team conducted the analysis, which first included familiarization with the data by reviewing the interview transcripts and making initial notes (Phase 1). Coders then identified and reviewed initial codes in the data (Phase 2). This was followed by the identification of codes and corresponding themes present in the data (Phase 3). Coders then refined codes and themes (Phase 4). Coders had meetings at each stage of analysis to discuss how themes should be named. At Phase 5, the lead researcher finalized and summarised the code and theme structure. Thematic analysis was conducted using Dedoose (Version 9.2.6; SocioCultural Research Consultants, LLC).

### Participants

The study was advertised through multiple channels: mothers and mothers‐to‐be support groups and internal newsletters at the first author's institution, the local council, the local National Childbirth Trust, and various social media group pages. Flyers were also posted across local venues. Study advertisements included a QR code to direct the mothers to an online account that we set up (Calendly, 2023) for mothers to pick a time that worked for them; some time slots were set up for individual interviews and some were set up for focus groups. In our study advertisements, we invited first‐time mothers with a child aged 3 years or younger who live in North East England (UK). This study was approved by Durham University's Psychology Ethics Committee.

We interviewed 22 mothers between the ages of 23 and 44 (*M* = 29.82, *SD* = 6.26); Their babies were between 2 and 34 months old^1^ (*M* = 16.15, *SD* = 8.61). The ethnic makeup of the sample was quite diverse. There were five British participants (i.e., English, Welsh, Scottish, Northern Irish), four identifying as other White backgrounds, three African participants, six identifying as other Black or Caribbean backgrounds, one Asian participant, and three identifying as other mixed or multiple ethnic backgrounds. Fourteen participants obtained undergraduate or bachelor's degrees whereas the rest had postgraduate degrees. Half of the sample were employed full time, four were employed part‐time, one was student, and six were unemployed. While most mothers were married (*n* = 15) and four were living with their partners, two participants in our sample were single mothers, and one was divorced. Most mothers were in good physical condition; three reported being in “excellent” health, 11 reported “very good”, and eight reported “good”. When asked about the amount of time they got to spend alone each week, five mothers said “definitely not enough”, eight said “somewhat less than [they] would like”, six said “just about right”, and only three said “somewhat more than [they] would like”.

### Results

Study 1 revealed a wide range of themes. Themes that are related more broadly to struggles during maternal transition, including changes in social relationships, feelings of guilt and internal pressure, and coping strategies, are not within the scope of this paper. They will be addressed more fully in a separate manuscript that is currently being prepared. Below, we focus specifically on themes related to mothers' experiences of aloneness.

### Theme 1: A baby's presence as an ambivalent type of companionship

At the beginning of each interview, participants were asked to define solitude in their own words. Interestingly, mothers described the presence of their baby as an ambiguous form of companionship. One mother reflected on this ambiguity, describing her time with her baby as being somewhere between solitude and social interaction. While the baby's presence is constant, it does not provide meaningful reciprocal interaction; which is often a key component of companionship.

Yeah, I think it's an interesting one because I spend most of my day with my son, my baby, he's 10 months old. Umm and he's there all the time but at the same time there is a level of solitude because he's not, like, it's not like somebody I can communicate with properly. (ID: 10)

When the demands of childcare become more salient, the presence of the baby can shift from being a reciprocal interaction to feeling like a series of tasks. The focus on feeding, washing bottles, and managing the baby's routine makes these moments feel more like responsibilities than true social engagement (see a review on what constitutes social contact in Schilbach et al., 2013). In such instances, the experience becomes less interactive and more isolating, leading mothers to feel as though they are alone even in the baby's presence.

I guess even though you are technically alone—well, my husband has gone back to work, so I'm alone with her through the day—but it doesn't feel alone in the same way. (ID: 35)

For some mothers, time spent at home with their baby did not qualify as solitude because the constant engagement with the baby's emotional and physical needs prevented opportunities for autonomy or personal relaxation. These mothers defined solitude as synonymous with “me‐time,” a period free from caregiving demands that allowed them to focus solely on themselves. By reserving the term solitude for “me‐time” moments, participants implied that time spent with the baby, while technically alone due to the absence of reciprocal communication, was not truly their own. In this sense, “me‐time” carried a more positive connotation, highlighting a distinction between simply being alone and having uninterrupted time for oneself. This perspective echoes prior research highlighting the important difference between solitude and me‐time (Rodriguez & Campbell, 2025).

So as much as spending time with my child is cute and sweet and all that, I wouldn't say it's part of my solitude time because, you know, I'll be engaging to make sure I am there for my child, like emotionally, play with them, feed them and all that. So I wouldn't include that in my solitude time. For me it's just my me‐time, you know. (ID: 3)

Another mother noted that her time spent with the baby—even during baby's sleep—did not allow for personal reflection or fulfilling activities. Instead, this time often felt like a continuation of her caregiving role, rather than an opportunity to disconnect or recharge.

So what I think I'd distinguish it into […] solitude that is just for me has not been natural or regular, […] so there has not been enough of that, no, but kind of enforced solitude during the day with the baby […] just grabbing whatever I can when they're asleep. (ID: 7)

As such, when time spent alone primarily involves tasks and chores, the baby's presence feels less fulfilling as an interaction. The nature of the mother's connection to her child can depend on the child's age and responsiveness, shaping how the mother perceives the baby's role in her solitude. The absence of reciprocal communication and the emphasis on caregiving needs can make the baby's presence feel more like an obligation than a meaningful interaction, leaving the mother in a state of ambivalent solitude. This echoes prior literature on subjective solitude, which suggests that it involves not just the absence of communication (Campbell & Ross, 2021) but also a detachment from social demands and obligations (Long et al., 2003; Nguyen et al., 2019). When solitude is overwhelmed with responsibilities, it becomes difficult to classify or experience it as truly restorative.

### Theme 2: Solitude during transition to motherhood is a multifaceted experience

When discussing the time alone not interacting with the baby, which would align more with the conventional conceptualization of solitude, mothers described their solitude as having different flavours depending on the activities they were doing and the choices they had in what they were doing. Within this theme, it becomes clear that mothers' experiences with solitude after becoming a mother reflect the challenges of balancing personal time with the demands of childcare.

#### Sub‐theme 1: Solitude as busyness

Several mothers shared the overwhelming experience of ongoing lists of household chores and responsibilities. Even during the brief moments of their child's naptime, mothers find themselves prioritising housework like cleaning, cooking, and laundry, since time feels scarce and unpredictable. Activities such as pumping and cleaning baby items add to the sense of a never‐ending cycle of tasks.

I was pumping because there was like this requirement of cleaning the bottles in a certain way and doing it non‐stop and cleaning the pump parts every single time—an unbelievable amount of teeny tiny fidgety things that have to be cleaned. […] So there was this I think that was like this constant question, I was taking away from my solitude. (ID: 39)

The opportunity to switch off and engage in rest or hobbies is rare, and the desire to do something for oneself is overshadowed by the pressing need to complete chores. This reflects a common sentiment among new mothers that any potential me‐time is consumed by necessary and time‐consuming tasks associated with running a household and caring for a baby.

When the baby naps, there is time to obviously do things, but I feel like I'm doing housework and as quickly as possible because they're gonna wake up at any minute. So I haven't had time to relax regularly or do anything that I want to do because I feel like there's so much that I have to do. (ID: 7)

I feel like now there is always a list of like jobs and things to do…so if anyone takes my little one out, I've got to finish up, gotta do washing up. There's just always something I don't even know what I would do to totally switch off now. (ID: 38)

And like when I was handling all the house chores and again the baby's there, I really have a lot in my hands such that I couldn't even spare some two minutes for myself. (ID: 69)

#### Sub‐theme 2: Solitude as “me time”

When mothers do have the liberty of having me‐time, which is often defined as time free away from childcare demands or chores, many mothers often prioritise basic self‐maintenance activities like napping or resting due to exhaustion. Showers can be a precious opportunity to switch off from the demands of motherhood, even if they are less frequent than desired.

It needs to be done I don't I don't really feel like I switch off. I switch off in the shower [laugh] It's probably my place now. I really appreciate a shower. […] But I don't get a shower often as I would like. I haven't had one today. (ID: 38)

Mothers also described opting for activities that require minimal effort and provide relaxation, such as listening to soothing music, as a strategy to recharge. These moments of personal activities provide breaks from the continuous responsibilities of parenting and help the mothers restore their energy.

I usually try at least to have at least thirty minutes or so of me‐time whereby just maybe take a long shower while listening to music or I just lie down and just appreciate that time for a moment. (ID: 12)

When you get that me‐time, I just really wish to get a nap for a few minutes and it's equally important when I'm not doing the things that I love doing, I gets myself to rest and sleep. (ID: 69)

#### Sub‐theme 3: Solitude as loneliness

During those solitary moments, loneliness can creep in. This is an often‐unspoken aspect of the transition to motherhood because why would a mother feel lonely when she gets to spend time with her baby all day? Besides the inability to have meaningful conversations with her small child, childcare responsibilities make it challenging for mothers to connect with friends or enjoy social activities as they once did. Mothers expressed missing the external energy and social interactions outside of the mundanity that comes with childcare and chores.

In that solitudeness, at that moment it come along with some element of loneliness. Well by yes you, you just went through embrace and appreciate that time, but you end up being lonely because you can't talk to this kid. You can't have a conversation with the kid. But again, you can't go out and hang with friends, have coffee, have those long calls. (ID: 69)

As such, loneliness can emerge because of the yearning for past social experiences that are now gone or significantly reduced or because of the absence of reciprocal interactions. Loneliness can also arise from the overwhelming demands and exhaustion. For example, one mother talked about those alone moments in the house, especially moments like late‐night feedings or when family support is unavailable.

I think that there is a loneliness even though you may have some time alone especially you're alone all the time in the middle of the night when you are breastfeeding … I mean you are nursing [but] … it's 3 o'clock in the morning and it's you; but I think that it's incredible the weight of the responsibility no matter if you have a partner, your mom is there, your sister, whoever, like it is on you like everything's happening. (ID: 39)

Difficult days, when things go wrong, can amplify feelings of isolation. The burden of being solely responsible for another human being, especially without immediate support, makes solitary moments more stressful and chaotic.

I have a happy baby compared to other babies, which is lovely, […] but yesterday he cried uncontrollably for an hour and I was really worried that something was really wrong. So when you're on your own, there's no one to, you know, check that that's normal or, you know, bounce ideas off. (ID: 7)

In attempts to overcome this feeling of loneliness, a few mothers developed strategies to engage in productive activities. For these mothers, productivity served not only as a distraction but also as a way to regain a sense of purpose and momentum. Rather than embracing solitude passively, they sought out tasks that would help them feel accomplished or occupied, particularly during periods of extended time alone.

I think for me solitude could be something that you can have too much of. And so for example kind of if my husband would go away for the weekend, the first thing kind of maybe half a day it would be great, you would do all the things that you wanted to do that you know, didn't have time to do when you were as a pair, […] but then, after a—maybe a day or so I would say I would kind of be a bit lonely and think oh—what have I got to do that I could do to occupy myself with […] to get that sense of accomplishment or something that you might get […] when you're doing it with someone. (ID: 35)

As such, participants often sought out things to do to create a sense of engagement and meaning in their day, sometimes including scheduled social interactions. In doing so, they became more intentional with their time, using productivity not just to stay busy but as a tool for emotional regulation. These structured activities offered a sense of stability and helped buffer against low mood.

Now during maternity leave, I make sure to have something scheduled in every day, or at least four out of the five days. Whether that's someone coming round or a class or a cup of tea to make to avoid any low feelings or enter to keep my mental health good because I know that I don't do well if I'm just on my own all day. (ID: 7)

### Theme 3: The changing nature of solitude after becoming a mother

Most first‐time mothers expressed that their sense of “solitude” significantly changed as the baby's needs became the central priority. They referred not to physical aloneness, which often increased, but to the subjective sense of being free from cognitive demands. The instinct to respond to every sound and movement of their child overrode opportunities for rest; even when partners were available to help.

In the first year maybe 9 months 10 months probably the first full year, even if I was alone there was always like this other—I can't read like a whole article and not think about my child […] Solitude in the first year you might get all sorts of solitude but for me like it wasn't mine and I was constantly thinking about the baby. (ID: 39)

This reduction in subjective solitude involved three key elements: shifting priorities, fragmented and unpredictable time, and reduced autonomy over how solitude was spent.

#### Sub‐theme 1: Shift in priorities reduces opportunities for me‐time

Mothers recognize the value of alone time and the benefits it could bring, yet they struggle to justify taking this time for themselves, as they did before motherhood.

I feel like I do value alone time and I would probably benefit from a bit more than I get now. But I feel like your priorities do adjust to, once you have a baby […] you don't put yourself first anymore, so I don't prioritise my alone time. It's their needs come first. So, I am not very good at taking the time for myself, where in the past I would've done that and not really thought anything of it if I just need some time out. But now I find it harder to justify for it. (ID: 38)

Priorities shift dramatically after one becomes a mother; self‐care activities that mothers used to enjoy, like going to the gym or going out, take a backseat to the child's needs. Even during rare moments of being free from childcare, the mother's thoughts are often still with the child; so even when they get to be alone, that solitude is still not entirely their own.

Before becoming a mother, one could more easily carve time out for herself as part of the daily routine that allowed for personal pursuits, hobbies, and relaxation. However, after childbirth, the constant needs of the baby take priority, leading to shorter, less frequent, and often more fragmented solitude.

I have shorter solitude time. Before I had like longer periods of my free time because you see now, with the baby and the baby needs a lot of attention and care, I have to like take care of her, like most of the time. Yeah. So the solitude is shorter and also it's less frequent. (ID: 87)

Even when external help is available and appreciated, family members who come to help care for the child can sometimes infringe further on mothers' ability to find time for themselves.

When I became a mother it was very jarring to go from (long pause) a decent amount of solitude […] and then all of sudden sort of overnight you're literally never alone and I don't, I never felt like I was alone even though [my] child isn't talking but they have needs, a lot of needs, uhm and then any kind of moment of alone time which I just didn't [have] … I mean my mother came for a month, my mother‐in‐law came for a month, my dad came for a month […] But it felt like really phrenetic, any moment of so—because I mean I was like I have to do all of these, I mean I have to get all of these things done…. (ID: 39)

#### Sub‐theme 2: Fragmented and unpredictable solitude

Because a mother's schedule is often dictated by the baby's needs, she has less control over the time she has for herself. Her child's naps might last, for example, 20 min or 90 min, and it is difficult to anticipate how much time she might have. This unpredictability makes planning difficult and often leads to frustration when mothers cannot complete tasks or enjoy any meaningful activities during these moments.

Initially, before I became a mom, I used to create that time and it was somehow easier cause I would just shut down my laptop, shut down my device […] but you know, currently this little human being that you are bringing up, you can't just ignore, maybe shut down like you could shut down the laptop, you just have to be present. […] what has made it difficult is generally the entire roles of being a mom. You have to feed this child, have to prepare the medicine, have to clean. Sometimes the baby can just decide today we're going to throw tantrums the entire day. And you know you have no control over that or the baby. (ID: 12)

Solitude often came by chance, with no guarantee of quality or duration. The inability to predict the length of these moments creates a sense of urgency and restricts the activities mothers feel they can engage in.

It's quite fragmented, […] he'll nap but that would be anything from like 20 minutes and like an hour, 90 minutes, even in rare cases. So like for that, you are a bit more limited in what you can do. Coz also you don't know how much time you have. Umm like it could be an hour, it could be half an hour and it is like broken up throughout the day. (ID: 10)

On days when the child is sick or not feeling well, this may significantly reduce the quality of these moments, leaving mothers feeling overwhelmed instead of recharged.

Sometimes the baby cries so it can be very challenging for me to have a peaceful environment … let's say that the baby is sick […] it would be very hard for me to spend my free time like … how I planned about it. (ID: 74)

Some mothers have adapted by seizing these moments opportunistically, engaging in activities that require minimal effort or can be paused easily. However, for others, the unpredictable nature of these intervals leads them to give up planning altogether, resulting in a sense of helplessness.

#### Sub‐theme 3: Loss of autonomy in solitude

The biggest change that mothers mentioned about the time alone they have after becoming a mother is the decreased autonomy over how they spend their solitude.

So I'm breastfeeding, so there's a lot of time where I just, there's nothing else I can do except breastfeed and so I thought I'd be able to text friends more, but […] it's much more involved than I thought it would be. So, the only thing I can do is either watch telly or listen to a podcast, I need something that I don't need my hands for. So it's been nice to catch up on series that I haven't watched, but that is more time sat down than I am used to. (ID: 7)

This loss of autonomy extends to the kinds of activities mothers can engage in during their rare moments of solitude. Hands‐free activities such as watching television or listening to podcasts are preferred, not out of personal choice, but because of practicality. Mothers' activities are typically confined to indoor spaces so that they can quickly respond to the baby's need. This limits mothers' opportunities to leave the house for social interaction, pursue activities that they used to enjoy (e.g., nature walks, cycling) and means that any activities may need to be paused or stopped at a moment's notice. Being confined inside the home also makes it difficult for mothers to mentally disengage from childcare, adding to their frustration.

And when I don't wanna read a book, I go play a guitar because it's in the house. And if the baby wakes up, I'm going to hear. So yeah, I just try to do things that are around the house […] the ones that are possible because of course I can't go cycling. I can't go for nature walks unless I have the people with me. (ID: 86)

For many mothers, solitude has shifted from being an intentional or self‐directed time to something restricted by caregiving responsibilities. This constrained autonomy also highlights the shift in how solitude is experienced. Post‐motherhood solitude becomes something reactive and functional, designed to fit around the unpredictable nature of childcare. Subsequently, reduction of choice and options in solitude, along with piled up responsibilities, undermine mothers' ability to enjoy this time, as previous research has shown autonomy and choice to be important factors that shape the quality of solitary experiences (Nguyen et al., 2018, 2019). These constraints can deepen the sense of isolation, as mothers are unable to engage in the types of hobbies or pastimes that might previously have brought them joy or connection to others.

## STUDY 2

Study 1 revealed the nuances of mothers' experiences with time spent alone, which increased significantly after having the first baby. The presence of an infant during that time blurs the line between solitude and social interaction, as some time might be bonding time if interactions are reciprocal, while other times it feels more solitary if filled with obligations and childcare demands. Outside of bonding time, solitude is either overwhelmed with responding to the baby's needs or lonely from the feelings of going at it alone. This makes solitude feel less like “me‐time” and mothers have little time to pursue their personal activities; if any, it would be maintenance activities such as making meals or taking showers.

Building on Study 1, Study 2 tracked mothers' activities during the transition to motherhood and looked at the associations of those activities with the mothers' emotional and psychological well‐being. Specifically, Study 2 focused ontime alone with baby, time alone without baby, time spent on chores, and personal time for baths, meals, and leisure activities.

### Recruitment

We recruited participants in the North East region of England through words of mouth, mother groups, and advertisements on local communities' Facebook pages. We were explicitly looking for first‐time mothers with a child younger than 3 years old. Again, the advertisements directed the mothers to an online calendar so they could pick a time that worked for them; we included availabilities over the weekend for full‐time working mothers.

### Procedure

When a mother signed up for a lab session, she received an email with an anonymous ID and a link to the demographics survey (i.e., see below for “Initial Survey”), hosted on Qualtrics (Version 2023; www.qualtrics.com). As the mother arrived at her scheduled session, she was instructed to engage in a 10‐min recorded free play with her baby and fill out a questionnaire, which she could opt out of if her baby was <8 months old. The free play and additional questionnaire were part of other studies and are therefore not included in the analysis of this paper.

We assessed mother's daily activity engagement and psychological experiences. To track this for the next seven days, we asked mothers to download the ExpiWell app (www.expiwell.com), which sent notifications for them to complete ecological momentary assessments (EMA) at five different times: 8 am, 11 am, 2 pm, 5 pm, 8 pm. The EMAs that went out between 8 am and 5 pm were available for 1 hr, and the one at 8 pm was open until midnight. We showed participants examples of the surveys so they could ask questions during the lab session before starting their EMAs the next morning. To encourage response rates, we compensated mothers incrementally depending on the number of surveys they completed: £4 for attending the in‐lab session and £3 for every 5 surveys completed afterward. After seven days of data collection, we sent mothers a debriefing form to ask for their feedback and compensate them. All our mothers reported being able to complete the surveys without any problems.

### Measurements

#### Initial survey

When participants signed up for the study, they were asked about their household's total yearly gross income, ethnicity, employment status, highest level of education, and marital status. Their child's age and gender were also reported. To assess each mother's psychological and physical states, we included measures of mothers' depressive symptoms, physical health, and life satisfaction. For depressive symptoms, we used the Center for Epidemiologic Studies Depression measure (CES‐D) (Radloff, 1977), which include items such as “I was bothered by things that usually don't bother me”, “I felt depressed”, or reverse‐coded items such as “I felt hopeful about the future”. Participants were asked to rate the items using four response options, “rarely or none of the time (less than 1 day)”, “some or a little of the time (1–2 days)”, “occasionally or a moderate amount of time (3 to 4 days)”, “most or all of the time (5–7 days)”. To measure mothers' general physical and psychological health, we used single items, “how do you rate your current physical health?” and “all things considered, how satisfied are you with your life these days?”. Finally, like Study 1, we asked mothers about the average number of hours they spent in solitude, and whether they felt that that amount was either not enough or too much.

#### Ecological momentary assessments

Three types of survey were set up on ExpiWell. The start‐of‐day survey was sent out at 8 am; three activity‐diary surveys were sent out between 11 am and 5 pm; finally, the end‐of‐day survey was sent out at 8 pm.

#### Start of day

##### Emotion

At the beginning of each day, mothers reported their current emotions. We included items representing emotions of four different dimensions: high‐arousal positive emotions (i.e., cheerful, excited), high‐arousal negative emotions (i.e., irritated, tense), low‐arousal positive emotions (i.e., calm, relaxed), low‐arousal negative emotions (i.e., bored, sad). These items were adapted from the Pick‐A‐Mood instrument (Desmet et al., 2016) and each item was accompanied by a picture representing that emotion. Participants rated the items on an 11‐point scale from 0 (not at all) to 10 (very much). The *multilevel.reliability* command from the ‘*psych*’ package in the R program showed that all four dimensions showed good reliability of the average of all ratings across all items and days (>.70).

##### Sleep quality

One item was used to assess participants' quality of sleep the previous night (Snyder et al., 2018). We used a scale ranging from 0 to 10, with 0 labelled “terrible”, 2 “poor”, 5 “fair”, 8 “good”, and 10 “excellent”.

##### Stress, loneliness, and boredom

Mothers' levels of stress, loneliness, and boredom at the beginning of the day were measured with three single items: “At the moment, I feel stressed/lonely/bored”. We asked mothers to respond to those items on scales from 0 (not at all) to 100 (very much).

To maximize our sample, we aggregated start‐of‐day mood, sleep, and ill‐being variables across days to calculate each participant's general baseline for these variables. This way, if a mother did not complete the start‐of‐day survey on a particular day, her data for that day could still be analysed by controlling for her baseline levels.

#### Activity diary

##### Activities

We asked participants what they were doing during each one‐hour interval in the past three hours. For example, the survey sent out at 11 am asked mothers about their activities between 8–9, 9–10, and 10 am–11 am. Participants first saw an open‐ended question where they could list the main activities, and then were asked to categorise those activities into any of the following types: (1) personal time (bath, reading, meal, cooking, exercise), (2) personal entertainment (gaming, YouTube, Netflix), (3) relaxation and resting, (4) social activities (family and friends), (5) work‐related activities (schoolwork, jobs, productive tasks, emails, etc.), (6) chores and errands, (7) volunteer or extracurricular activities, (8) care responsibilities (childcare, care for a family member, etc.), or none of the above. Those categories were adapted from the activity codes used in Time‐Use Surveys (Office for National Statistics, 2023). If a category was chosen for that hour, it got a code of 1; each participant was instructed that they could choose as many categories as apply. For example, if a mother spent the hour between 9 and 10 am having breakfast, taking care of her baby, and socialising, she may categorise this hour into three categories (1, 4, 8 in the list above). The scores for each activity throughout the day were averaged across hours to yield the percentages of time each mother engaged in each category of activity out of all the surveys she responded to on that day.

##### Aloneness

To assess how frequently the mother spent time alone, we asked her about who she was with: adults living in the same household, child or children up to 3, child or children aged 3+ to 18, relatives, friends or colleagues, strangers, with a crowd. We also asked whether the mother was interacting with anyone during that hour, including interactions that happened on the phone, online, or on social media. If the mother reported being only around “strangers” or “with a crowd” but not engaging in any social interaction, the incident was counted as “all alone.” If she reported being around familiar others, such as family, relatives, friends, or children older than 3, the incident was coded as “interaction.” Based on the mother's responses, we categorized each incident as one of the following: (1) “all alone” (i.e., alone without the baby, may be around strangers or in a crowd but not interacting), (2) “alone with baby” (i.e., alone except for the baby and not interacting with anyone else remotely), or (3) “interacting with others” (i.e., in the presence of familiar others). The scores for these two variables throughout the day were also averaged across hours to yield the percentages of time that each mother spent all alone (i.e., ‘all alone’) or alone with the baby (i.e., ‘alone with child’) out of all the surveys she responded to on that day.

As such, Study's operationalization of solitude builds on insights from Study 1, whereby we distinguish between solitude with and without the presence of the baby. Importantly, it captures subjective solitude, as situations in which our participants were surrounded by strangers or a crowd of people (except for familiar others and the baby) were still treated as instances of “all alone” solitude. Consistent with the literature (Campbell & Ross, 2021; Nguyen & Rodriguez, 2024), this operationalization reflects perceived detachment from social demands (such as from familiar others), including both physical and non‐physical (e.g., virtual or online) interactions.

##### Location

We also asked the mother where she was during that hour, whether at home, at work or place of education, public space, or whether she was travelling. There was also an open‐ended “other” option. We recoded this item into a variable that represents being at home. The scores for this variable throughout the day were then averaged across hours to yield the percentage of time that each mother spent at home (i.e, ‘at home’) out of all the surveys she responded to on that day.

#### End of day

The mothers were asked again to report their activities in the past three hours. Additionally, the same emotion items were also included, and we assessed levels of stress, loneliness, and boredom that mothers experienced at the end of the day. To measure stress, we used the short‐form 4‐item Perceived Stress Scale (Ingram et al., 2016; Vallejo et al., 2018); an example item was “Today, how much have you felt you were unable to control the important things in your life?”. To measure loneliness, we used the 3‐item UCLA Loneliness Scale (Hughes et al., 2004); an example item was “Today, how much do you feel that you lack companionship?”. Finally, to measure boredom, we used the 7‐item Multidimensional State Boredom Scale (Hunter et al., 2016); two example items were “Today, I felt bored” and “Today, I was wasting time that would be better spent on something else”. The participants responded to those three scales on 5‐point scales from 0 (not at all) to 4 (very much).

### Sample

Our sample consisted of 47 new first‐time mothers; none of the mothers from Study 1 was in the Study 2 sample. We ran a simulated power analysis (1000 simulations) and determined that a sample of 47 with 7 data points per person (totalling up to 329 daily observations) would allow us to detect an effect size as small as *R* ^2^ = .04 with more than 80% power. Overall, the majority of our participants provided data for all 7 days (*n* = 42), with 3 participants completing 6 days, 1 completing 4 days, and 1 completing 3 days. So in total, we obtained 319 daily observations from the mothers in our sample; this was 10 observations (3%) short of what we calculated our power for, which would have minimal impact on power. As such, we proceeded with *R* ^2^ = .04 as our smallest effect size of interest (SESOI). All our participants in Study 2 were Caucasians and between the ages of 24 and 42 (*M* = 31.91, *SD* = 3.99). Most mothers were from households that earned annual incomes of more than £52,000 (*n* = 32), and most had either an undergraduate degree or a postgraduate qualification (*n* = 42). There was no single mother in the Study 2 sample; 31 mothers reported being married, and 16 were living with their partners. Their babies were between 1 and 33 months old (*M* = 10.89, *SD* = 7.18); there were relatively equal numbers of baby boys (*n* = 23) and girls (*n* = 24).

This was an affluent sample of first‐time mothers and were mostly in good physical condition; most of our mothers reported being in either “good” health (*n* = 25), or “very good” to “excellent” health (*n* = 19), and only three reported “poor” physical health. On a 5‐point single item asking about mothers' life satisfaction, the median was 4. Regarding the phenomenon of interest—mothers' perceptions of how much solitude they had, 18 of the participants said that their amount of solitude was “just about right” while 25 said it was either “somewhat less than they would like” or “definitely” not enough. Only four said that they had somewhat more solitude than they would like. We also did not have any mothers that experienced severe depressive symptoms (*M* = 1.54, *SD* = .35; range = [1.00, 2.55]).

### Data analysis

We conducted linear mixed effect models using the ‘lmer’ function from the package ‘lme4’ (Bates et al., 2015) in the R software (RStudio Team, 2023). We were interested in which activities that mothers spent more time performing on a given day predicted their emotional experiences, stress, loneliness and boredom reported at the end of that day. We first controlled for mothers' depressive symptoms, the child's age, the baseline level of the outcome variable (measured at the beginning of the day), and previous night's quality of sleep. Then we added the variables ‘at home’, ‘all alone’, and ‘alone with child’, followed by the eight activity categories. With the sample size of 319 data points across 47 mothers, we did not include random effects for all the predictors; instead, we included only the person‐level and day‐level random intercepts.

## RESULTS

### Descriptive statistics

**FIGURE 1:**
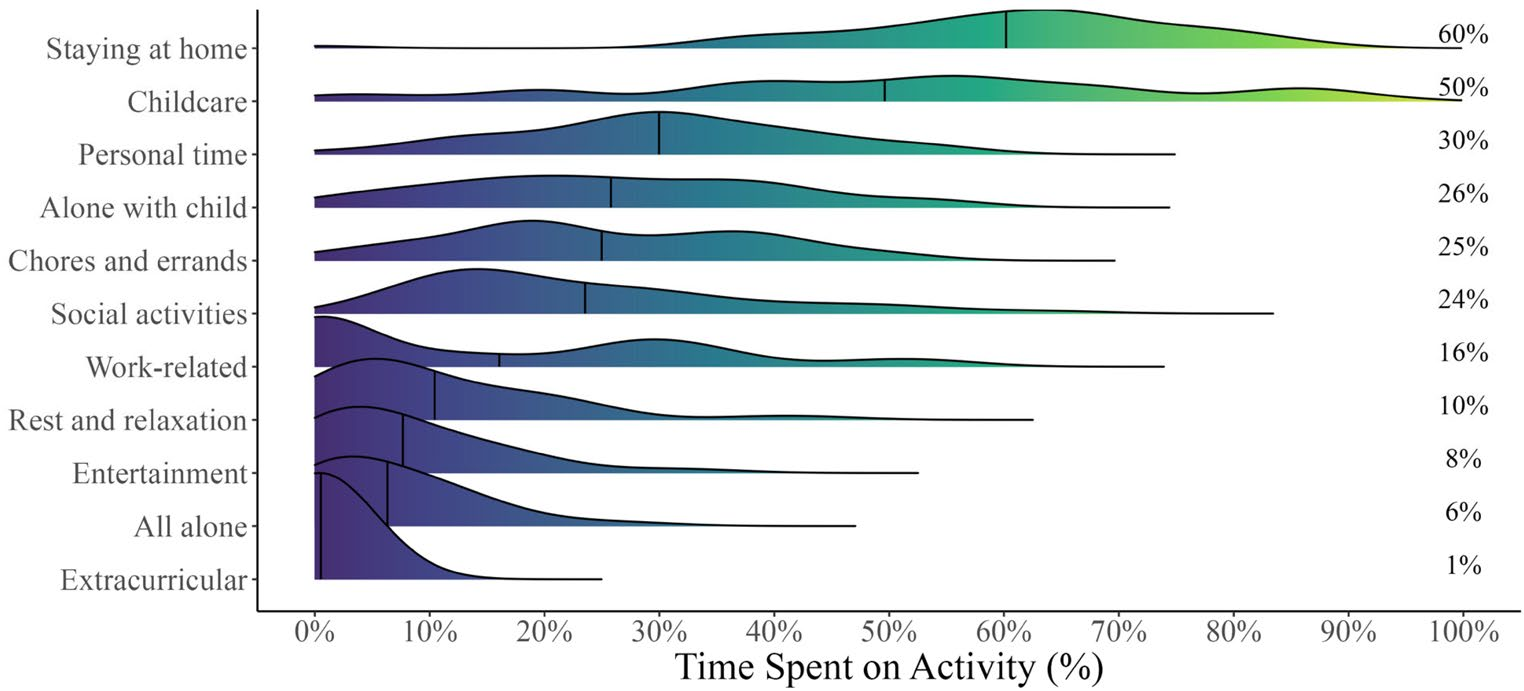
Distribution of time spent on daily activities (%). Numbers on the right represent the mean percentage of time spent on each activity.

We present the average percentages of time engaged in each activity category in Figure 1. On average, our sample of mothers spent most of their day staying at home (60%) and engaging in childcare (50%). Time spent alone with the baby on average took up 26% of the day, but there was very little time that the mother spent all by herself (6%). There were relatively similar proportions of time dedicated to personal time (30%), chores and errands (25%), and social activities (24%). The most popular activities for these mothers' personal time included quality time with the baby, making or eating meals, watching television, taking a walk, enjoying a cup of tea, or self‐maintenance activities like getting dressed or taking a shower. Because most of the mothers also worked (24 full‐time and 13 part‐time), there was some time dedicated to work‐related activities (16%), and even less time for rest and relaxation (10%), followed by time for personal entertainment (8%) and very little time for extracurricular activities like going to baby or swim lessons (1%).

Time spent on childcare varied across mothers, with 50% of the variance that can be attributed to individual‐level differences. Aside from that, time spent on other activities varied less, with variance due to individual differences ranging between 7% and 27%. Analyses of intraclass correlation coefficients suggested moderate to high reliabilities in the differences observed between individuals (ranging between .60 and .87) for most activities (see Table 1).

**TABLE 1:** Within‐group correlations between activity categories.

Variable	1	2	3	4	5	6	7	8	9	10	ICC
1. Personal activities											.23
2. Entertainment	.01										.27
3. Rest and relaxation	.05	.18***									.32
4. Social time	−.07**	−.08***	−.05								.26
5. Work‐related tasks	−.26***	−.10***	−.12***	−.17***							.26
6. Chores and errands	−.04	−.08***	−.10***	−.22***	−.17***						.23
7. Extracurricular activities	−.05	−.03	−.03	.00	−.04	−.02					.07
8. Childcare	.01	−.03	−.07**	−.06*	−.32***	.02	.04				.50
9. All alone	.06*	.02	.05	−.12*	.05	.03	−.03	−.23***			.26
10. Alone with child	−.02	.06*	.00	−.28***	−.14***	.08***	−.03	.29***	−.16***		.20
11. Stay at home	.17***	.19***	.16***	−.29***	−.20***	.08***	−.07*	.24***	−.07**	.24***	.19

*Note*: Significance levels are adjusted with Holm corrections.

Abbreviation: ICC, intraclass coefficients.

* *p* < .05;

** *p* < .01;

*** *p* < .001.

Table 1 presents within‐person correlations between categories of activities, including time spent alone (with or without the baby) and time spent at home. Being completely alone (All Alone) is positively associated with staying at home (*r* = .29), suggesting that solitude often occurs within the home environment. While All Alone shows weak positive correlations with personal activities (*r* = .06), being alone with the child (Alone with Child) is either negatively or uncorrelated. This indicates that moments of complete solitude, without the presence of the child, may provide opportunities for self‐care or relaxation, but this is not always guaranteed. In contrast, time spent Alone with Child is linked to childcare (*r* = .29) and chores (*r* = .09), while showing negative correlations with social time (*r* = −.28) and work‐related tasks (*r* = −.14). These findings suggest that time spent alone with the child often competes with more externally oriented activities, such as socializing or work. These patterns align with Study's results, indicating that time spent alone at home with a young baby frequently involves caregiving responsibilities, while only moments of complete solitude allow mothers to focus on self‐care.

Start‐of‐day mood, sleep quality, and ill‐being levels (i.e., loneliness, stress, boredom) showed little to no association with activity engagement across the day (see Supporting Information). We found only three significant correlations. On days when mothers felt more excited and cheerful (*r* = .34), or calm and relaxed (*r* = .22), they engaged more in social activities. On the other hand, when they felt less calm and relaxed at the start of the day, they tended to do more work‐related tasks (*r* = −.21). So, while we found little evidence that how mothers felt at the beginning of the day shaped their activity engagement, we still controlled for their overall baseline levels of these experiences in our models predicting end‐of‐day outcomes.

When we looked at end‐of‐day variables, we found that mothers' positive emotions fluctuate from day to day, with 28% of high‐arousal positive emotions and 27% of low‐arousal positive emotions explained by differences between mothers. Negative emotions show higher stability over time, with 47% of high‐arousal negative emotions and 43% of low‐arousal negative emotions that can be explained by individual differences. When it comes to daily reports of stress (ICC = .38), loneliness (ICC = .31), and boredom (ICC = .38), there were also some fluctuations across days. Altogether, those intraclass correlation coefficients indicate that, while all emotional and psychological well‐being outcomes suggest some levels of stability (between 20% and 50% of variance explained by individual‐level differences), there is still significant within‐person change. As such, this provides further support for our next analyses to investigate how mothers' engagement in daily activities contributed to the daily fluctuation of emotions and well‐being. For every outcome, we controlled for baseline levels measured at the beginning of the day, as well as individual levels of depressive symptoms, sleep quality, and the child's age. These control variables were selected to account for differences between mothers.

#### Primary analyses

We entered all activities to linear mixed effect models to predict first‐time mothers' daily emotions, stress, loneliness, and boredom levels. All outcome variables assessed at the end of the day (after 8 pm) were most strongly predicted by the baseline levels measured at the beginning of the day (between 8 and 9 am). Examining individual differences between mothers, we found that mothers' baseline depressive symptoms were predictive of several negative outcomes, including lower calmness and relaxation (*B* = −1.26, *SE*(*B*) = .59, *β* = − .18 [−.35, −.01], *p* = .033, *R* ^2^_partial_ = .04), more experiences of stress (*B* = .64, *SE*(*B*) = .24, *β* = .27 [.07, −.46], *p* = .008, *R* ^2^_partial_ = .07), greater loneliness (*B* = .49, *SE*(*B*) = .19, *β* = .23 [.05, .41], *p* = .011, *R* ^2^_partial_ = .05), and more boredom (*B* = .40, *SE*(*B*) = .19, *β* = .20 [.01, .39], *p* = .039, *R* ^2^_partial_ = .04). Baby's age appears to be a small factor linking to mother's daily emotions; those who have younger babies tend to report more daily feelings of irritation and tenseness (*B* = −.05, *SE*(*B*) = .02, *β* = −.16 [−.30, −.03], *p* = .016, *R* ^2^_partial_ = .03).

##### Emotions

**FIGURE 2:**
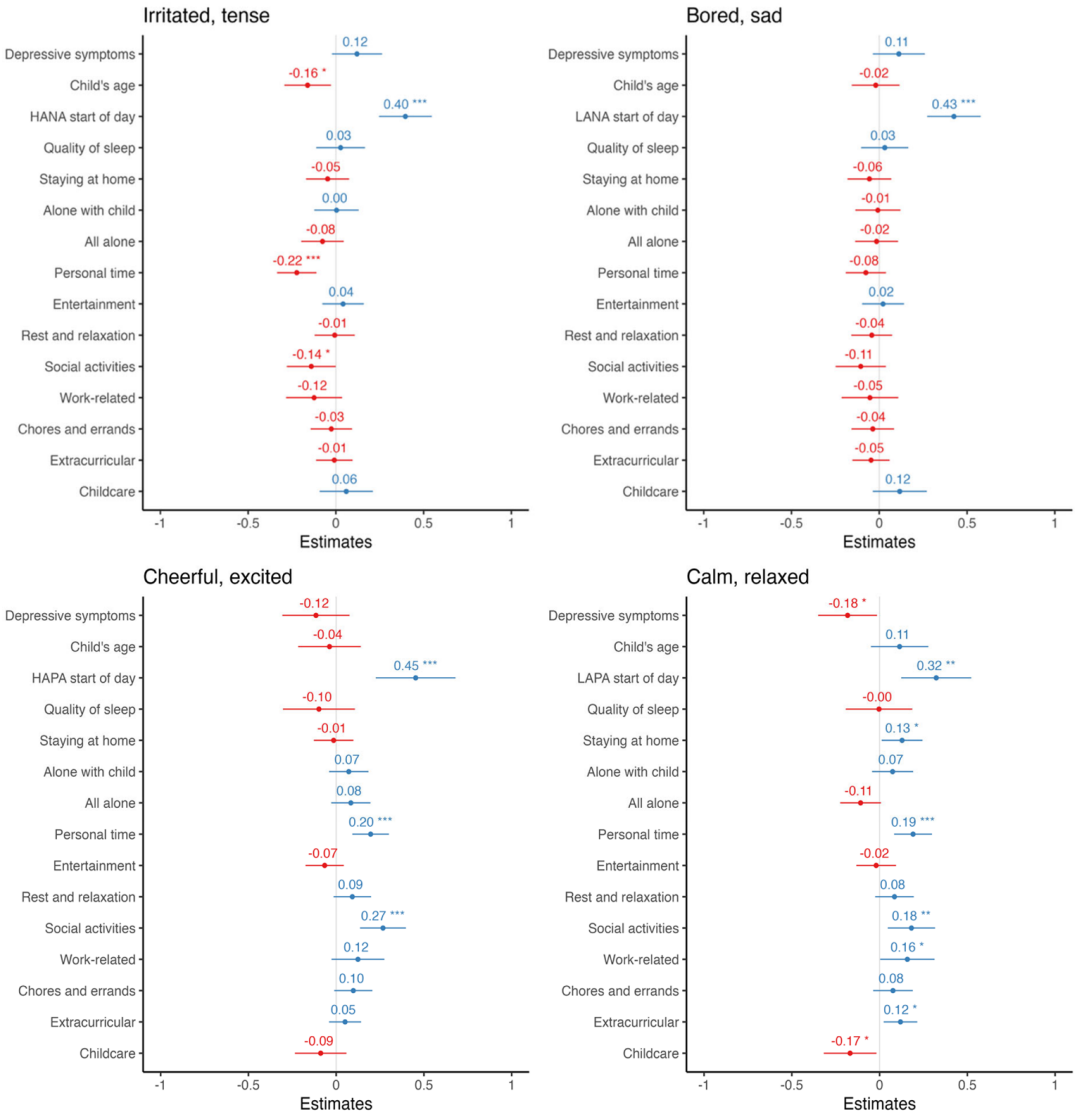
Plotting the coefficient of each predictor variable (*y*‐axis) and daily emotions (negative emotions at top, positive emotions at bottom) in *lmer* models. *p < .05, **p < .01, ***p < .001. Outcome variables (top label) were measured at the end of the day.

The results showed that personal time and social activities were the two most reliable predictors of mothers' daily emotions (see Figure 2). Having personal time positively predicted positive emotions like cheerful, excited (*B* = 2.06, *SE*(*B*) = .56, *β* = .19 [.09, .30], *p* < .001, *R* ^2^_partial_ = .06) as well as calm, and relaxed (*B* = 2.15, *SE*(*B*) = .62, *β* = .19 [.08, .30], *p* = .001, *R* ^2^_partial_ = .05). On the other hand, less personal time was associated with being irritated and tense (*B* = −2.12, *SE*(*B*) = .55, *β* = − .22 [−.33, −.11], *p* < .001, *R* ^2^_partial_ = .06). Spending more time with friends and family were also associated with more positive emotions (cheerful, excited: *B* = 2.59, *SE*(*B*) = .65, *β* = .27 [.14, .40], *p* < .001, *R* ^2^_partial_ = .07; calm, relaxed: *B* = 1.86, *SE*(*B*) = .70, *β* = .18 [.05, .32], *p* = .008, *R* ^2^_partial_ = .03) and fewer negative emotions (irritated, tense: *B* = −1.25, *SE*(*B*) = .62, *β* = − .14 [−.28, −.00], *p* = .044, *R* ^2^_partial_ = .02).

Calmness and relaxation were also predicted positively by spending time at home (*B* = 1.20, *SE*(*B*) = .55, *β* = .13 [.01, .24], *p* = .031, *R* ^2^_partial_ = .02), performing work‐related activities (*B* = 1.33, *SE*(*B*) = .66, *β* = .16 [.00, .31], *p* = .045, *R* ^2^_partial_ = .02), or engaging in extracurricular activities such as going to gym or baby classes (*B* = 10.37, *SE*(*B*) = 4.24, *β* = .12 [.02, .21], *p* = .015, *R* ^2^_partial_ = .02). Performing care responsibilities negatively predicted calmness and relaxation (*B* = −1.28, *SE*(*B*) = .58, *β* = − .17 [−.32, −.02], *p* = .028, *R* ^2^_partial_ = .02). Interestingly, doing chores positively predicted feelings of being cheerful and excited (*B* = 1.24, *SE*(*B*) = .61, *β* = .11 [.00, .22], *p* = .044, *R* ^2^_partial_ = .02).

##### Stress, loneliness, boredom

**FIGURE 3:**
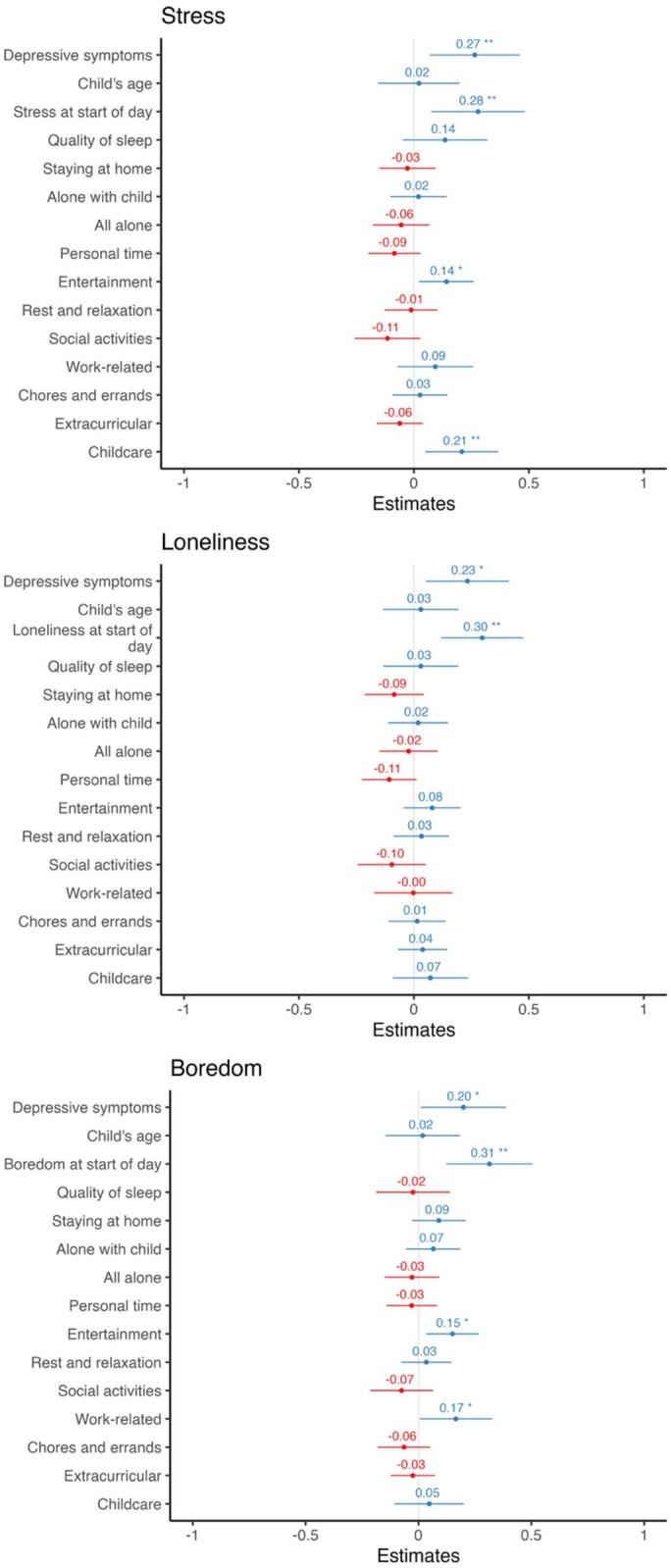
Plotting the coefficient of each predictor variable (*y*‐axis) in *lmer* models. *p < .05, **p < .01, ***p < .001. Outcome variables (top label) were measured at the end of the day.

For these three outcomes, as mentioned above, mothers' depressive symptoms consistently played an important role in explaining daily stress. Above and beyond this individual difference, personal time and social activities did not significantly predict first‐time mothers' daily levels of stress, loneliness, nor boredom. Surprisingly, spending more time on personal entertainment such as watching Netflix or YouTube was associated with greater stress (*B* = .94, *SE*(*B*) = .40, *β* = .14 [.02, .26], *p* = .020, *R* ^2^_partial_ = .03) and boredom (*B* = .83, *SE*(*B*) = .32, β = .15 [.03, .27], *p* = .011, *R* ^2^_partial_ = .03). Mothers' daily stress levels on a certain day were also linked to increase in childcare responsibilities on that day (*B* = .56, *SE*(*B*) = .22, *β* = .21 [.05, .37], *p* = .010, *R* ^2^_partial_ = .03) (see Figure 3).

**FIGURE 4:**
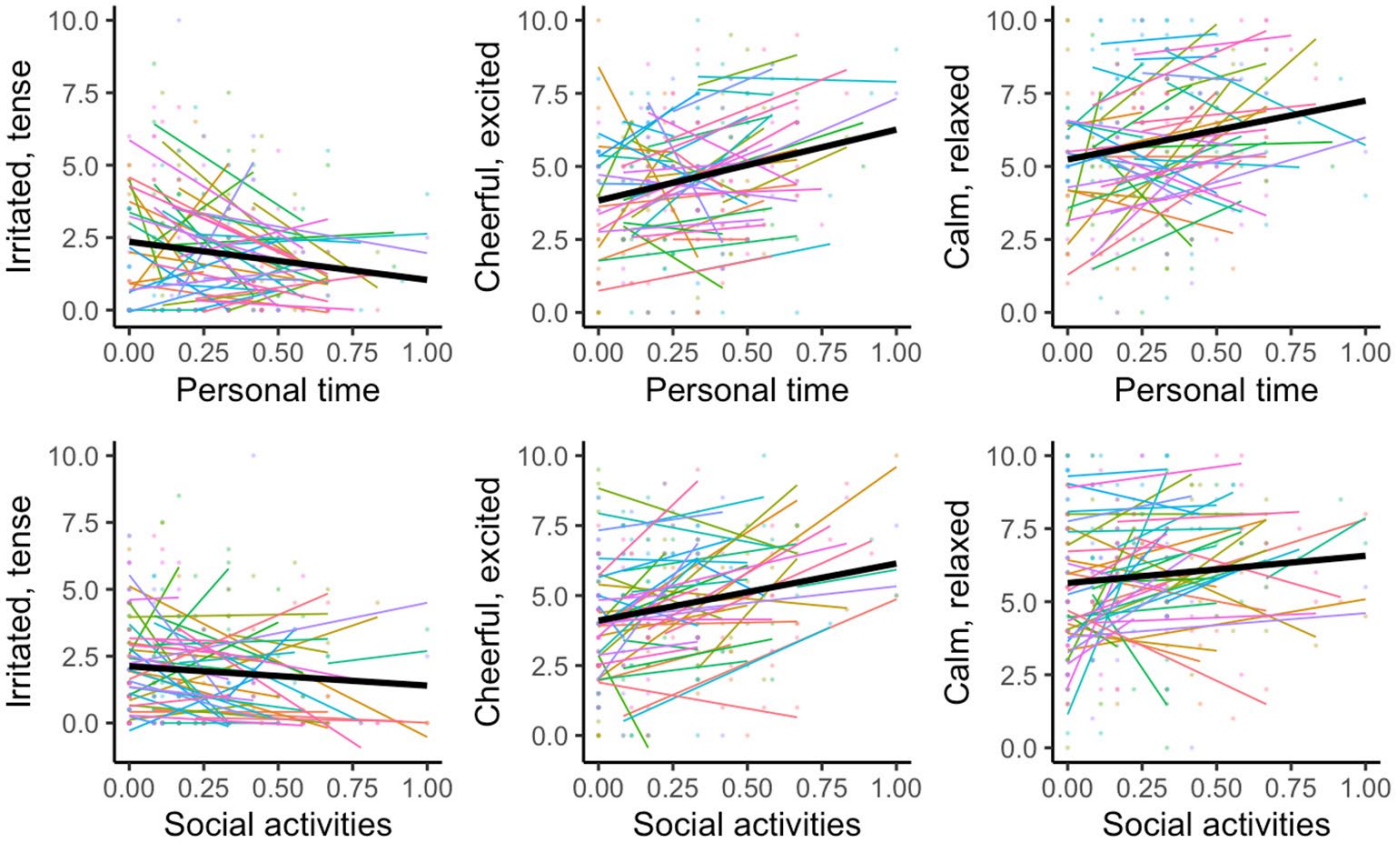
Scatterplots showing relationships between personal time and social activities with emotion outcomes for each mother.

Overall, having personal time was the strongest predictor of first‐time mothers' daily positive emotions. As discussed earlier, the sample size for this study allowed us more than 80% power to detect an effect size as small as 0.04. All the effect sizes we found for personal time linking to emotion outcomes were above 0.04. Another category of activities that was linked to daily positive emotions was time interacting with friends and family. When we graphed the correlations between personal time and social activities with the emotion outcomes that they predicted individually for each mother (see Figure 4), we saw that the effects were consistent across most mothers in our sample, with some mothers showing stronger effects than others.

## DISCUSSION

Previous research highlights “aloneness” as a common experience during the transition to motherhood. This study explored the nuanced nature of this phenomenon among two samples of British first‐time mothers. Study 1 revealed that mothers distinguish between being alone without the baby and being alone with the baby. The level of intimacy and reciprocity in interactions with the baby shapes whether this time is perceived as solitude. When alone time with the baby is dominated by tasks, chores, and childcare demands, it feels less like meaningful companionship and more like responsibility.

For many mothers, solitude is rarely time for self‐care; instead, it is consumed by childcare and household tasks, which often take precedence over basic needs like eating or showering. Limited opportunities for self‐care and social engagement can exacerbate feelings of loneliness, particularly for mothers who have babies with health problems, mothers who do not get enough family or friend support, or single mothers.

Study 2 supported these findings with quantitative data, showing that childcare consumed the largest portion of mothers' days, with chores and errands occupying 25%. Even moments of downtime between feeds or during naps were often filled with tasks, perpetuating a sense of never‐ending busyness. This fragmentation of time left little room for meaningful self‐care, which was typically reduced to basic necessities like sleeping, eating, or showering, as discovered in Study 1. When we included time spent on all the activities in the same model, we found that personal time consistently predicted improved daily mood; above and beyond the benefits of social interactions on those outcomes.

It was interesting that having more personal time on a given day was linked to more positive emotions and lower tenseness. However, personal time did not predict feelings of boredom, stress, or loneliness. For those outcomes, pre‐existing depressive symptoms were the strongest predictors, suggesting that such feelings may reflect more persistent psychological challenges rather than being shaped by daily activities. These findings highlight the importance of addressing underlying mental health concerns alongside day‐to‐day stressors in supporting new mothers.

Entertainment, often limited to passive activities like watching television or listening to music, was chosen for its compatibility with multitasking (e.g., breastfeeding). However, these activities were not associated with improved mood. Instead, they were linked to stress and boredom, suggesting that they might either result from or contribute to these negative feelings. It is plausible that spending more time engaging in media‐related activities may make mothers more stressed and bored because they did not have other activity options. Alternatively, daily stress and boredom could lead mothers to engage more in those activities. Our design did not allow us to determine the directions of those associations. Nonetheless, these findings suggest that what mothers considered to be their personal time might be different from the time they spent watching entertainment programs.

Overall, the lack of rest, scarcity of personal time, restricted social engagement, and overwhelming childcare and household responsibilities appear to contribute to mothers' negative daily moods. Personal time showed a small but significant positive correlation with time spent alone without the baby (*r* = .06), but negative correlations with social time (*r* = −.07) and time for work‐related tasks (*r* = −.26). These patterns suggest that new mothers tend to carve out personal time when they are away from both the baby and others, and when they are not engaged in work activities like answering emails or writing reports.

While these findings are situated within the context of new motherhood, they may also be relevant to other life circumstances that share similar demands, such as high‐stress jobs where individuals are constantly on call or cognitively occupied. In such environments, sustained external demands along with minimal opportunities for restorative time may also increase vulnerability to loneliness, isolation, and emotional exhaustion. These circumstances highlight that the experience of “aloneness” may be shaped not only by physical separation from others (i.e., physical solitude) or social disconnectedness, but also by the desperate need for personally meaningful time.

As such, our findings further expand the current understanding of subjective solitude, typically conceptualized as a state of disengagement and detachment from social contact and demands (Long et al., 2003). While previous work has speculated that solitude may still be experienced in the presence of others (Long et al., 2003; Nguyen & Rodriguez, 2024), our study is the first to provide empirical evidence of this in the context of early motherhood. We show that another person's presence, such as a baby's, does not necessarily take away the experience of being alone. Even when no communication takes place between the mother and the baby (Campbell & Ross, 2021), the meaning of the baby's presence depends on the nature of the demands it brings. These demands, in turn, shape how solitude is experienced and how it affects emotional well‐being, pointing to new directions for research: the quality of solitude may not only hinge on physical separation and absence of social contacts (Nguyen & Rodriguez, 2024), but also on the extent to which one can mentally disengage.

## LIMITATIONS

This research was the first attempt to dissect the complexity of “aloneness” experience often described in the literature on becoming a mother. To achieve the rich data collected from interviews with first‐time mothers as well as data of mothers' daily activities, this research relied entirely on convenience sampling from local communities. Therefore, the biggest limitation of our studies was the lack of sample diversity, which might have failed to capture a wide range of experiences of first‐time mothers from other health and demographic profiles. For example, mothers' ages and socioeconomic statuses are important risk factors for postpartum depression, and services available to depressed mothers vary depending on their racial backgrounds (Guintivano et al., 2018). Levels of risks associated with mothers' socioeconomic backgrounds also vary between countries, with those in developing countries suffering from greater risks (Norhayati et al., 2015). Levels of support were the main explanation for elevated risks for these groups and likely play an important role for first‐time mothers in our studies to find time for basic self‐maintenance activities with ease.

Additionally, although the EMA approach in Study 2 offered insights into mothers' daily routines and emotional experiences, the repeated‐survey method may pose practical challenges. During the debrief process, a few participants commented that the timing of prompts was quite rigid and difficult for them to manage, especially early in the morning when they had caregiving and work responsibilities. As such, while we did not face high rates of attrition or missing data, the study design would make it challenging for mothers who face time constraints to participate, which may introduce a potential bias toward more affluent or well‐resourced participants. Future research could explore more flexible and inclusive designs and focus on what types and levels of support new mothers need to meaningfully access time alone.

Another limitation that we recognise in this research is the lack of the partner's perspectives. Previous literature suggested that the non child‐bearing partner, commonly the father, who does not face the physical challenges of childbirth, is also significantly impacted by this transition, suffering from negative outcomes like stress, deprivation of both sleep (Saxbe et al., 2018) and me‐time (Levesque et al., 2020). What is more important is how parenting partners navigate tensions and conflicts in their relationships during this transition to find balance between childcare, personal time, and “us” time (Levesque et al., 2020). As such, we do recognise that accounting for both parents' perspectives is crucial.

## CONCLUSION

In summary, this research sheds light on how the experience of “aloneness” manifests during the transition to becoming a mother. The findings highlight the struggle with constant childcare demands and a scarcity of time for basic self‐care, which may contribute to daily negative experiences. With never‐ending cycles of chores and constant pressures to attend to the needs of their child, mothers' subjective experiences of time alone are often burdensome rather than restorative. Notably, personal time, although scarce, unpredictable, and fragmented, emerged as a crucial factor for enhancing mothers' daily mood. The findings from both studies emphasised that quality time for mothers to attend to their own needs can be just as important as social interactions and is more influential in mitigating the emotional challenges of motherhood than previously recognised. These insights call for more nuanced support structures that address these specific needs of first‐time mothers, ensuring they have access to both social support and opportunities for meaningful personal time to ease the demands of becoming a mother.

## AUTHOR CONTRIBUTIONS

**Thuy‐vy Nguyen:** Conceptualization; methodology; data curation; formal analysis; supervision; funding acquisition; visualization; writing – original draft; writing – review and editing; investigation; software. **Delali Konu:** Data curation; formal analysis; project administration; writing – review and editing; writing – original draft. **Deborah Tetteh:** Data curation; formal analysis; writing – review and editing. **Pearl Tshimbalanga:** Data curation; writing – review and editing; formal analysis. **Julie Weissova:** Data curation; formal analysis; writing – review and editing. **Mingyao Xiong:** Data curation; formal analysis; writing – review and editing.

## FUNDING INFORMATION

This research was funded by the Economic and Social Research Council (Ref. ES/W002256/1).

## CONFLICT OF INTEREST STATEMENT

There are no conflicts of interest to declare.

## Supporting information


Data S1.

## Data Availability

The data that support the findings of this study are openly available in OSF at https://osf.io/7jk9h/?view_only=7980fba9d0824d3b920a78660ad09a2b.

## Footnotes

^1^
Data on baby's sex was not collected in Study 1; but was added in Study 2.
