# Supplementary material for: “I got all sorts of solitude, but that solitude wasn't mine”: A mixed‐methods approach to understanding aloneness during becoming a mother
Source: Br J Psychol. 2025 Sep 2;117(1):192–216. doi: 10.1111/bjop.70019 (PMC12783874; doi:10.1111/bjop.70019)
Supplement: Supplementary file 1 — Data S1. [file BJOP-117-192-s001.docx]

**Supplementary material**

*Within-Group Correlations Between Start-of-Day Sleep, Mood, and Ill-Being and Activity Engagement*

| Variable | Sleep | HANA | HAPA | LANA | LAPA | Loneliness | Stress | Boredom |
| --- | --- | --- | --- | --- | --- | --- | --- | --- |
| 1 - Personal activities | 0.06 | -0.13 | 0.10 | -0.04 | 0.07 | -0.03 | -0.14 | 0.10 |
| 2 - Entertainment | -0.08 | -0.09 | -0.09 | 0.07 | 0.10 | -0.06 | -0.05 | -0.04 |
| 3 - Rest and relaxation | 0.06 | -0.02 | 0.11 | -0.01 | 0.17 | 0.02 | -0.19 | 0.16 |
| 4 - Social time | -0.03 | -0.09 | 0.34*** | -0.11 | 0.22* | -0.07 | -0.08 | -0.11 |
| 5 - Work-related tasks | 0.13 | 0.12 | -0.18 | 0.06 | -0.21* | -0.04 | 0.14 | -0.04 |
| 6 - Chores and errands | -0.10 | -0.04 | 0.02 | -0.13 | 0.02 | 0.01 | -0.02 | 0.02 |
| 7 - Extracurricular activities | -0.09 | -0.01 | 0.09 | -0.04 | 0.03 | -0.02 | -0.03 | -0.07 |
| 8 - Childcare | -0.10 | -0.03 | -0.01 | 0.04 | 0.00 | 0.05 | -0.04 | 0.08 |
| 9 - All alone | 0.06 | -0.01 | -0.09 | 0.06 | -0.03 | 0.06 | -0.10 | 0.17 |
| 10 - Alone with child | -0.01 | 0.06 | -0.09 | -0.02 | -0.10 | -0.02 | -0.02 | 0.07 |
| 11 - Stay at home | 0.03 | -0.08 | -0.05 | -0.01 | 0.07 | 0.01 | -0.12 | 0.08 |
| Notes. * *p* < .05, ** *p* < .01, *** *p* < .001. *Significance levels are adjusted with Holm corrections* | | | | | | | | |
